# Publisher Correction: Abrupt perturbation and delayed recovery of the vaginal ecosystem following childbirth

**DOI:** 10.1038/s41467-024-46160-8

**Published:** 2024-02-26

**Authors:** Elizabeth K. Costello, Daniel B. DiGiulio, Anna Robaczewska, Laura Symul, Ronald J. Wong, Gary M. Shaw, David K. Stevenson, Susan P. Holmes, Douglas S. Kwon, David A. Relman

**Affiliations:** 1 Department of Medicine, Stanford University School of Medicine, Stanford, CA 94305 USA; 2 Department of Statistics, Stanford University, Stanford, CA 94305 USA; 3 Department of Pediatrics, Stanford University School of Medicine, Stanford, CA 94305 USA; 4 Ragon Institute of MGH, MIT, and Harvard, Cambridge, MA 02139 USA; 5 Division of Infectious Diseases, Massachusetts General Hospital, Boston, MA 02114 USA; 6 Department of Microbiology & Immunology, Stanford University School of Medicine, Stanford, CA 94305 USA; 7 Section of Infectious Diseases, Veterans Affairs Palo Alto Health Care System, Palo Alto, CA 94304 USA

**Keywords:** Microbiome, Preclinical research

Correction to: *Nature Communications* 10.1038/s41467-023-39849-9, published online 12 July 2023

The original version of this Article contained an error in Figure 3b, where the Greek symbol letters were missing from the end of the cytokine names. This has been corrected in both the PDF and HTML versions of the Article.

