# Supplementary material for: Abrupt perturbation and delayed recovery of the vaginal ecosystem following childbirth
Source: Nat Commun. 2023 Jul 12;14:4141. doi: 10.1038/s41467-023-39849-9 (PMC10338445; doi:10.1038/s41467-023-39849-9)
Supplement: Supplementary file 1 — Supplementary Information [file 41467_2023_39849_MOESM1_ESM.pdf]

SUPPLEMENTARY INFORMATION

Abrupt perturbation and delayed recovery of the vaginal ecosystem following childbirth

Elizabeth K. Costello[1*], Daniel B. DiGiulio[1], Anna Robaczewska[1], Laura Symul[2], Ronald J. Wong[3], Gary M. Shaw[3], David K. Stevenson[3], Susan P. Holmes[2], Douglas S. Kwon[4,5], David A. Relman[1,6,7*]

[1]Department of Medicine, Stanford University School of Medicine, Stanford, CA 94305, USA
[2]Department of Statistics, Stanford University, Stanford, CA 94305, USA
[3]Department of Pediatrics, Stanford University School of Medicine, Stanford, CA 94305, USA
[4]Ragon Institute of MGH, MIT, and Harvard, Cambridge, MA 02139, USA
[5]Division of Infectious Diseases, Massachusetts General Hospital, Boston, MA 02114, USA
[6]Department of Microbiology & Immunology, Stanford University School of Medicine, Stanford, CA 94305, USA
[7]Section of Infectious Diseases, Veterans Affairs Palo Alto Health Care System, Palo Alto, CA 94304, USA

This PDF contains Supplementary Figures 1-9 and Supplementary Tables 1-4.

Supplementary Figures

Supplementary Figure 1: Collection timelines for vaginal swabs analyzed in this study.

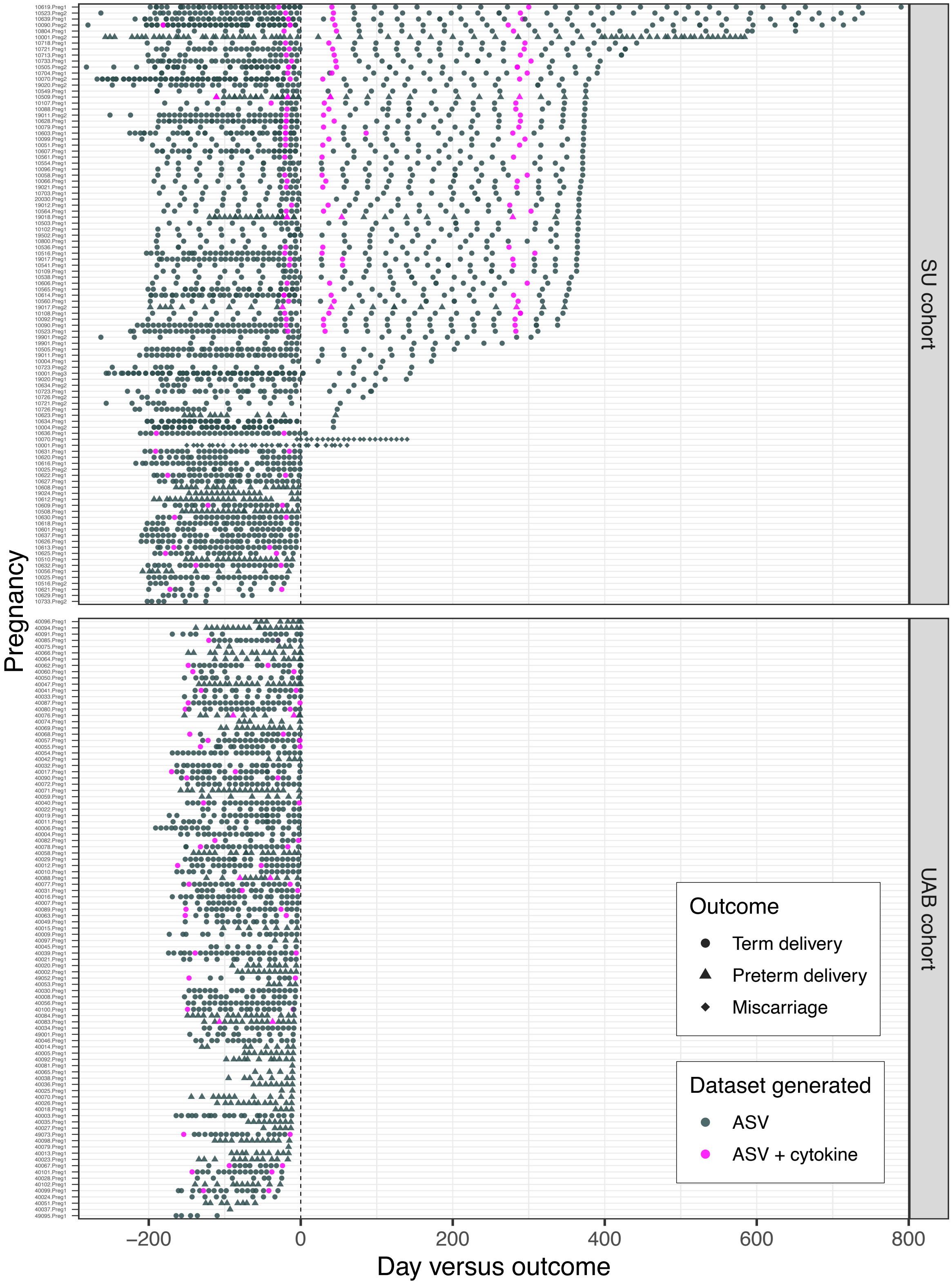

Supplementary Figure 1: Collection timelines for vaginal swabs analyzed in this study.

Pregnancies appear on the y-axis with labels indicating the participant and enrollment (Preg1, Preg2, or Preg3). The x-axis represents time relative to the end of gestation, which is marked by a vertical dashed line. Timepoint shape corresponds to the event that occurred on day zero (circles, term delivery; triangles, preterm delivery; diamonds, miscarriage). For each timepoint (n = 3,848 unique vaginal swabs), 16S rRNA genes were amplified, sequenced, and resolved to ASVs. For each timepoint in magenta, a paired swab was analyzed for cytokine/chemokine concentrations (n = 198 unique vaginal swabs). Exactly overlapping (i.e., entirely opaque) timepoints indicate samples for which ASVs were derived in technical duplicate (see Methods). Pregnancies with postpartum sampling appear at top (SU cohort, n = 72 pregnancies, 40 with cytokine/chemokine analysis), followed by those without postpartum sampling (SU cohort, n = 28 pregnancies, 9 with cytokine/chemokine analysis; UAB cohort, n = 96 pregnancies, 29 with cytokine/chemokine analysis).

Supplementary Figure 2: Temporal changes in the diversity and composition of vaginal bacterial communities are uncoordinated with the progression of gestation.

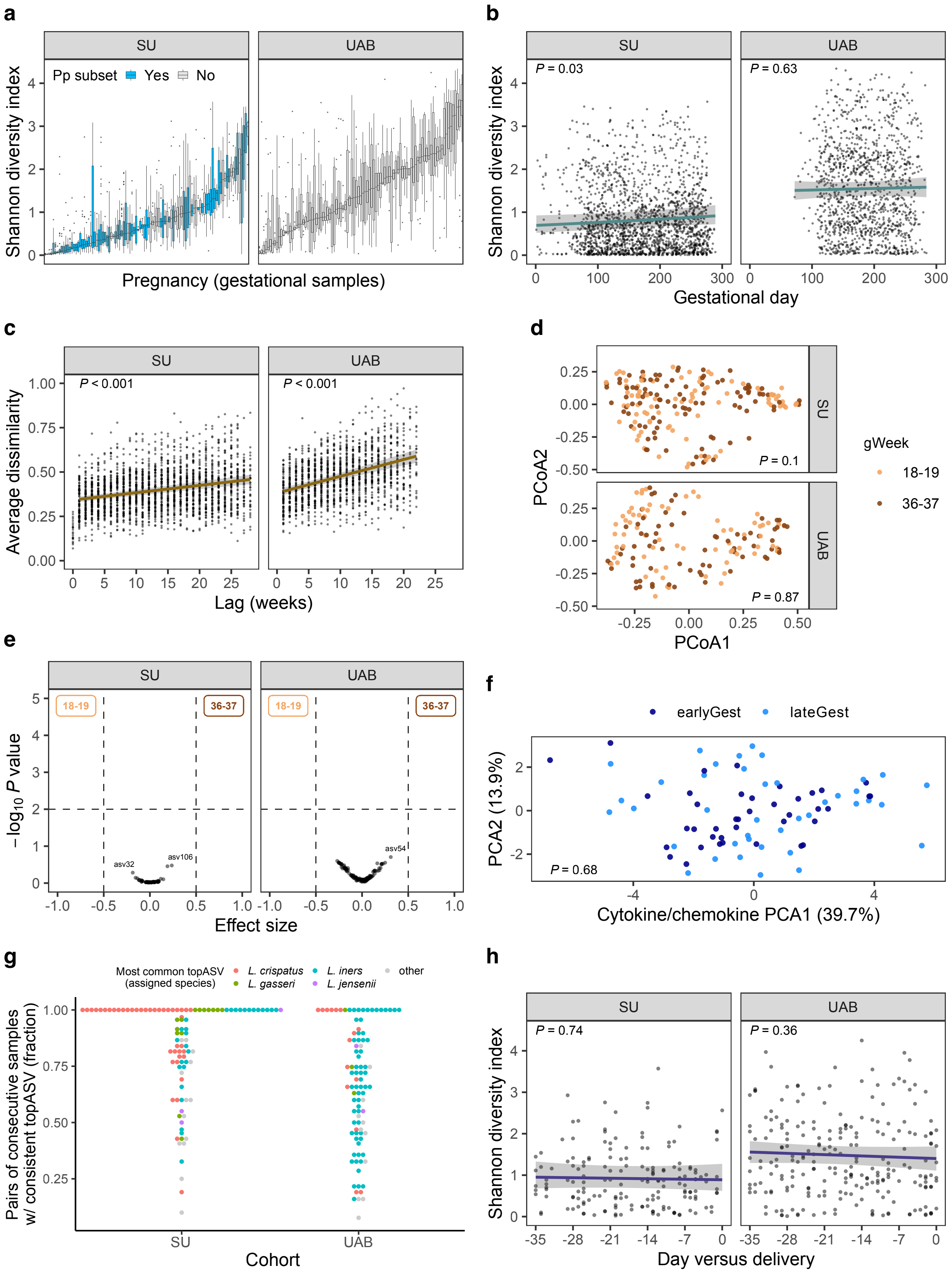

Supplementary Figure 2: Temporal changes in the diversity and composition of vaginal bacterial communities are uncoordinated with the progression of gestation.

a, Boxplots summarizing the distribution of the Shannon diversity index, a measure of alpha diversity, over each gestation. Pregnancies in blue are those that went on to collect postpartum (Pp) samples. Four pregnancies, each with only one gestational sample available for analysis, were omitted from this and subsequent panels. Left facet, SU cohort; right facet, UAB cohort. Boxplots depict the median, IQR (hinges), and most extreme values within 1.5 * IQR of the hinges (whiskers).

In **b,** the Shannon diversity index is plotted against gestational day. Points correspond to samples. Lines indicate linear mixed-effects (lme) regressions and shaded areas represent 95% confidence intervals. Per-pregnancy slopes and intercepts were modeled as random effects. SU cohort, marginal R^2 = 0.002, conditional R^2 = 0.757, n = 99 pregnancies; UAB cohort, marginal R^2 < 0.001, conditional R^2 = 0.620, n = 93 pregnancies. (The relatively high conditional R^2 values suggest that much of the unexplained variation in the Shannon diversity index is associated with differences between pregnancies.) Controlling for gestational day, vaginal bacterial communities were significantly more diverse in UAB participants than in SU participants (P < 0.001, n = 192 pregnancies; lme model augmented with Cohort as a fixed-effect term and applied to the combined dataset). Congruent results were obtained using Faith's phylogenetic diversity (PD) as the alpha diversity metric. Significance was evaluated by t-test.

In **c,** the average Bray-Curtis dissimilarity, a measure of beta diversity, is plotted against temporal lag in weeks. Position along the vertical axis corresponds to an average of pairwise dissimilarities between same-pregnancy, same-lag samples. Pairwise lags were calculated as the absolute difference (in days) and binned to the week. Linear-mixed effects modeling was performed as in panel **b**. SU cohort, marginal R^2 = 0.072, conditional R^2 = 0.699, n = 99 pregnancies; UAB cohort, marginal R^2 = 0.165, conditional R^2 = 0.777, n = 93 pregnancies. The rate of temporal turnover was significantly higher in UAB participants than in SU participants (P < 0.001, n = 192 pregnancies; lme model augmented with Cohort as a fixed-effect interaction term and applied to the combined dataset). Congruent results were obtained using weighted UniFrac as the distance metric. Bray-Curtis dissimilarities and weighted UniFrac distances were calculated using fourth root transformed count data to moderate the influence of highly dominant ASVs. Points at Lag = 0 represent pairs of technical replicates and were not included in the model. Significance was evaluated by t-test.

d, Ordination of samples collected early and late in gestation. "Early" was defined as gestational weeks 18-19 (early-to-mid 2nd trimester) and "late" was defined as gestational weeks 36-37 (mid-to-late 3rd trimester) giving a lag of approximately 18 weeks of gestation. Pregnancies lacking a sample in either group were excluded from this analysis. For each cohort, Bray-Curtis dissimilarities were subjected to Principal Coordinates Analysis (PCoA) and permutation-based tests for differences in group dispersions and locations, none of which were significant (displayed P-values correspond to permutational MANOVAs, i.e., the location tests). SU cohort, n = 48 pregnancies, 89 early vs. 99 late samples, PCoA1 (22.3%), PCoA2 (13.3%); UAB cohort, n = 44 pregnancies, 70

early vs. 70 late samples, PCoA1 (25.4%), PCoA2 (16%). Congruent results were obtained using weighted UniFrac as the distance metric. Count data were transformed as in panel **c**.

e, Analysis of ASV differential (relative) abundance in comparisons of early versus late gestation. Shown are volcano-like plots in which the $-\log_{10}$ of the expected Benjamini-Hochberg corrected *P*-value (Wilcoxon tests) is plotted against the effect size, as calculated using the R package ALDEx2. Samples were identical to those analyzed in panel **d**. Per cohort, ASVs appearing in ≥ 15% of samples were analyzed (SU cohort, *n* = 74 ASVs; UAB cohort, *n* = 130 ASVs). ALDEx2 applies a centered log-ratio (clr) transformation to the count data. Dashed lines indicate our choice of cut-off values (absolute effect size > 0.5; corrected *P* value < 0.01). In each cohort, for the comparison of early versus late gestation, none of the tested ASVs were identified as differentially abundant. Congruent results were obtained using rank-based tests.

f, Ordination based on the cytokine/chemokine content of vaginal swabs collected early and late in gestation. PCA was applied to the log-transformed concentrations of 16 cytokines/chemokines measured in vaginal swab eluates (*n* = 80 unique samples) from 40 pregnant participants (SU cohort, *n* = 11; UAB cohort, *n* = 29). Analytes were IFN-γ, IL-1α, IL-1β, IL-5, IL-6, IL-10, IL-17, IL-21, IL-23, IP-10, ITAC, MIG, MIP-1α, MIP-1β, MIP-3α and TNFα, none of which differed significantly in concentration between the two gestational phases (two-sided Wilcoxon signed rank tests). The displayed *P* value corresponds to a permutational MANOVA using Canberra distances.

g, Dot plot depicting the fraction of consecutive gestational samples in which the identity (sequence label) of the top ASV remained the same. Each dot corresponds to a pregnancy (SU cohort, *n* = 99; UAB cohort, *n* = 93; a sample's top ASV is its most abundant ASV). The y-axis bin width is 0.02. Color indicates the taxonomy (species-level label) of the top ASV that occurred most frequently. For example, the purple dot at y = 1 represents a maximally stable pregnancy in which the identity of the top ASV (asv4 *Lactobacillus jensenii*) remained constant throughout. This measure of stability was significantly higher among SU pregnancies than among UAB pregnancies (two-sided Wilcoxon rank sum test; *P* < 0.001) and was correlated with dominance (Spearman's rho = 0.72; *P* < 0.001; dominance was defined as the per-pregnancy mean relative abundance of the top ASV, regardless of identity, across all gestational samples).

In **h,** the Shannon diversity index is plotted against day relative to delivery for the final five weeks of gestation in pregnancies with spontaneous onset of labor. Linear-mixed effects modeling was performed as in panel **b**. SU cohort, marginal R^2 < 0.001, conditional R^2 = 0.585, *n* = 36 pregnancies; UAB cohort, marginal R^2 = 0.004, conditional R^2 = 0.675, *n* = 60 pregnancies. Congruent results were obtained using Faith's PD as the alpha diversity metric. Significance was evaluated by t-test.

Supplementary Figure 3: Pregnant women's vaginal bacterial communities examined through the lens of each sample's most abundant ASV.

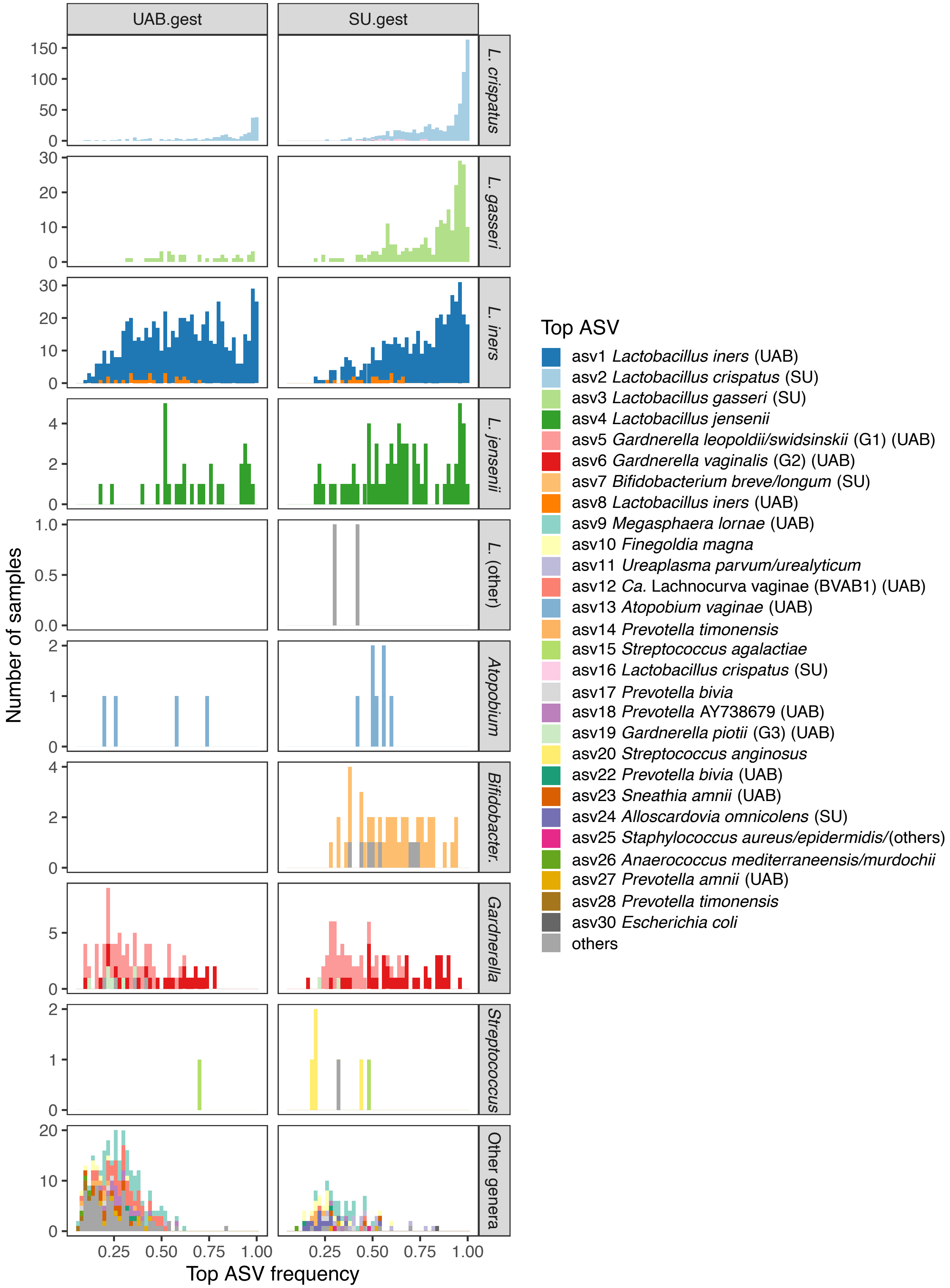

Supplementary Figure 3: Pregnant women's vaginal bacterial communities examined through the lens of each sample's most abundant ASV.

Histograms of top ASV frequency, which is defined as the relative abundance of the most abundant ASV in each sample and is inherently correlated with the Shannon diversity index (Spearman's rho = -0.96). Samples are colored by the identity of the top ASV and faceted into rows by the taxonomy of the top ASV. The x-axis bin width is 0.02. Note that the y-axis scale differs among taxa. Twenty-eight of the 30 most abundant ASVs study-wide, listed at right, appear as a top ASV in a sample collected during pregnancy. These 28 ASVs account for the top ASV in 96% of the depicted samples, in which they represent 88% of the total reads. Note that states of extreme dominance are held almost exclusively by *Lactobacillus* species. Left facet, UAB cohort, $n = 96$ pregnancies, 1276 unique samples displayed; right facet, SU cohort, $n = 99$ pregnancies, 1750 unique samples displayed. For each of the 28 ASVs, we tested for an effect of Cohort on the average gestational frequency (the mean frequency across all unique samples from the same gestation). Those annotated in the legend were significantly more abundant in the given cohort (two-sided Wilcoxon rank sum tests; cohort indicated if effect size (r) > 0.2 and Benjamini-Hochberg-adjusted $P < 0.01$; $n = 99$ SU pregnancies versus $n = 96$ UAB pregnancies). The largest effect size ($r = 0.9$) was observed for asv12, *Candidatus* Lachnocurva vaginae (BVAB1), which was present at > 0.1% relative abundance in 32% of UAB samples versus 0.5% of SU samples.

Supplementary Figure 4: Identification of delivery-responsive vaginal bacterial taxa.

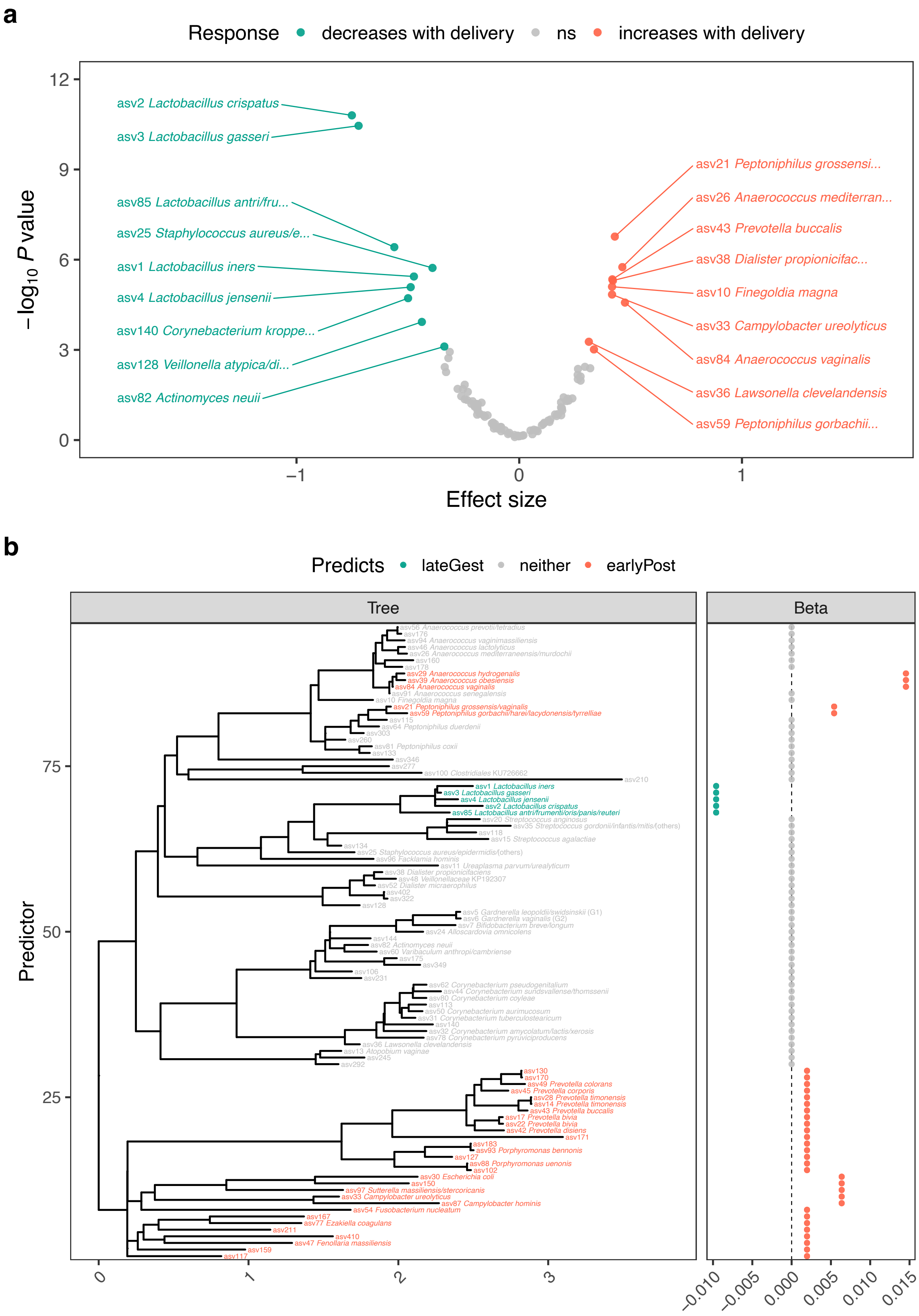

Supplementary Figure 4: Identification of delivery-responsive vaginal bacterial taxa.

a, Analysis of ASV differential (relative) abundance in a comparison of vaginal samples collected up to two weeks before delivery, to those collected up to 10 weeks after delivery (SU cohort, n = 65 pregnancies with timepoints in range, n = 102 lateGest samples versus 107 earlyPost samples). ASVs appearing in ≥ 15% of samples were analyzed (n = 96 ASVs, representing 88% of the reads in these samples). Shown is a volcano-like plot in which the $-\log_{10}$ of the expected Benjamini-Hochberg corrected P-value (Wilcoxon tests) is plotted against the effect size. The analysis was performed using the R package ALDEx2, which applies a centered log-ratio (clr) transformation to the count data. Corrected P-values < 0.001 were considered significant.

b, Results of tree-based sparse discriminant analysis, a supervised method. Leaf predictors (ASVs; left panel) are aligned with their regression coefficients (Beta; right panel) and colored according to the class they predict. Conservative cross-validation suggested 5 predictors (nodes in the tree) corresponding to 39 leaves. Count data were asinh-transformed prior to analysis; otherwise, input samples and ASVs were identical to those in panel **a**. Complete annotation labels are provided for ASVs in the top 100 most abundant study-wide.

Supplementary Figure 5: Delivery is associated with a vaginal pro-inflammatory cytokine response.

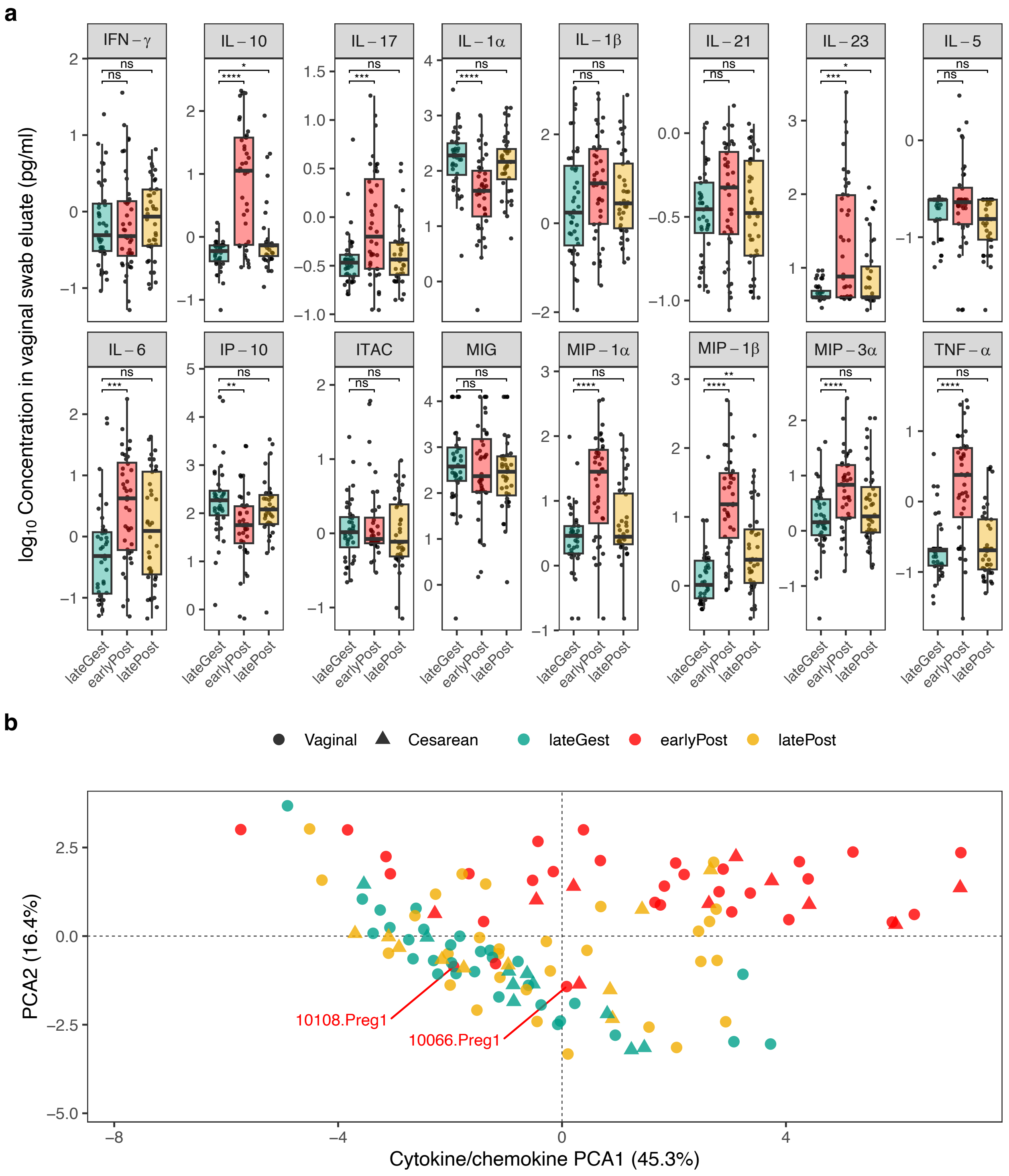

Supplementary Figure 5: Delivery is associated with a vaginal pro-inflammatory cytokine response.

a, Jitter plots depicting the $\log_{10}$-transformed concentrations of 16 cytokines/chemokines in eluates from vaginal swabs collected before and after delivery (SU cohort, n = 40 pregnancies). Boxplots summarize the data for each timeframe: ~3 weeks before (lateGest, green), ~6 weeks after (earlyPost, red), and ~9.5 months after delivery

(latePost, yellow). Significance levels correspond to Benjamini-Hochberg-adjusted P values for paired, two-sided Wilcoxon signed rank tests in which late gestation served as the reference timeframe. ****, $P < 0.0001$; ***, $P < 0.001$; **, $P < 0.01$; *, $P < 0.05$; ns, not statistically significant. Boxplots depict the median, IQR (hinges), and most extreme values within 1.5 * IQR of the hinges (whiskers).

b, PCA based on the data plotted in panel **a**. Color and shape indicate timeframe and delivery mode, respectively. Early postpartum samples from two pregnancies are labeled; these (and only these) two individuals remained *Lactobacillus crispatus*-dominated at their first postpartum sample (Fig. 2c). The effect of delivery on the cytokine/chemokine milieu was tested using permutational MANOVA based on Canberra distances with late gestation (lateGest) serving as the reference timeframe (earlyPost, $P < 0.001$; latePost, $P = 0.048$).

Supplementary Figure 6: Cohort-specific compositional signatures are retained across delivery.

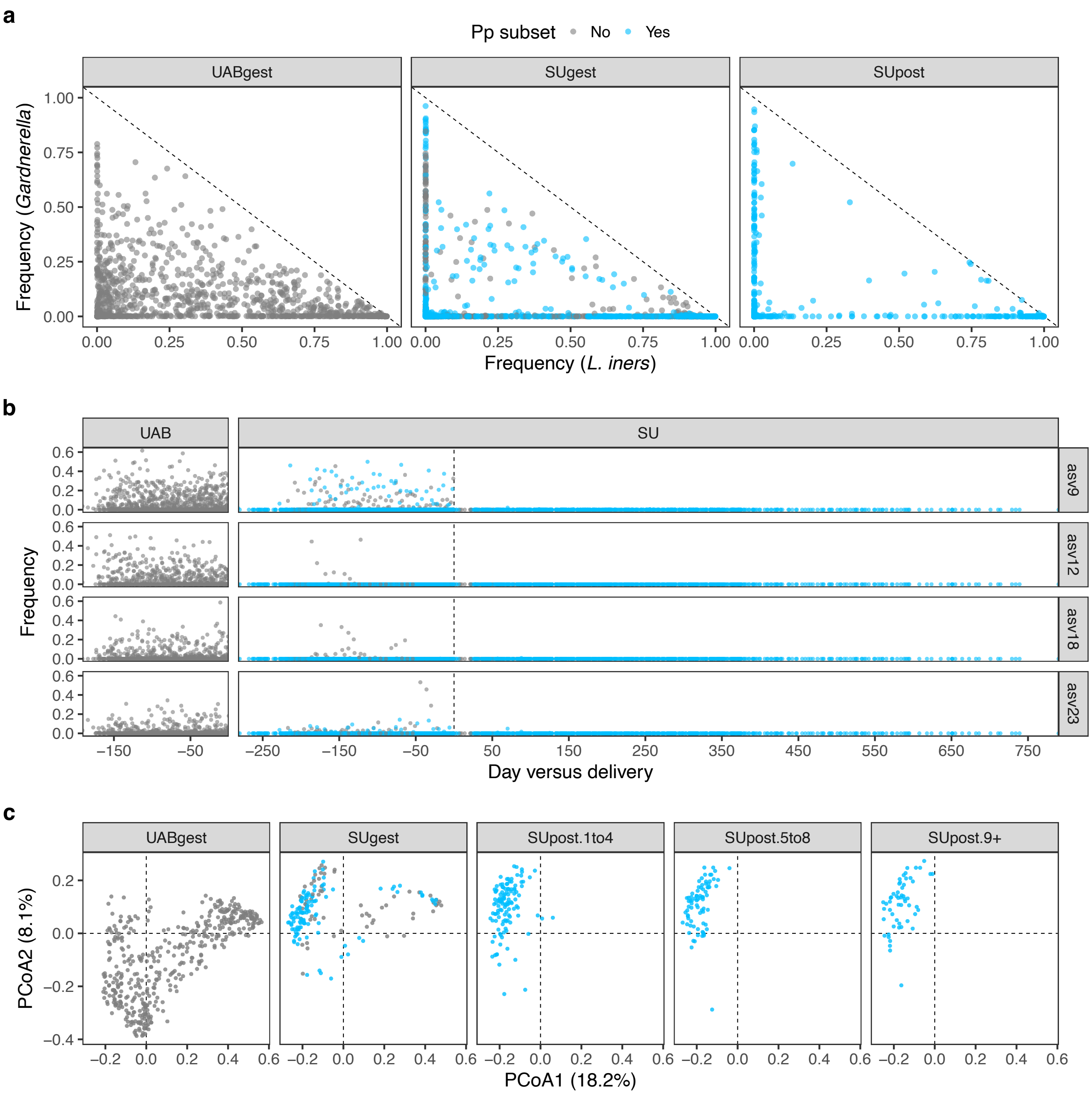

Supplementary Figure 6: Cohort-specific compositional signatures are retained across delivery.

a, *Gardnerella* frequency plotted against *Lactobacillus iners* frequency for vaginal swabs collected from the UAB cohort (left, while pregnant, n = 1276 samples) and from the SU cohort (center, while pregnant, n = 1750 samples; right, postpartum, n = 745 samples). Postpartum (Pp) subset indicated in blue. *Gardnerella* and *L. iners* often co-occur at comparable frequencies in the UAB cohort (left facet) – a signature that was not acquired by the SU cohort after they delivered (right facet).

b, Frequencies of four ASVs – asv9 (*Megasphaera lornae*), asv12 [*Ca*. Lachnocurva vaginae (BVAB1)], asv18 (uncultivated *Prevotella* sp.), and asv23 (*Sneathia amnii*) – plotted over time. These ASVs often occur at high frequency in the UAB cohort. This signature was not acquired post-delivery by the SU cohort.

c, Ordination of diverse vaginal microbiota, defined as having a Shannon diversity index > 2 (n = 880 samples). The ordination method was Principal Coordinates Analysis (PCoA) and the dissimilarity metric was binary Jaccard, which is presence-absence based and therefore emphasizes community membership. ASVs were filtered to those with at least 20 reads in at least 10 samples (n = 801 ASVs). A single ordination is plotted, with samples facetted by cohort and timing (e.g., "SUpost.1to4" refers to samples collected 1-4 months after delivery). Diverse postpartum membership appears to track diverse gestational membership in a cohort-consistent manner.

Supplementary Figure 7: Pre- and post-delivery vaginal bacterial communities viewed through the lens of each sample's most abundant ASV.

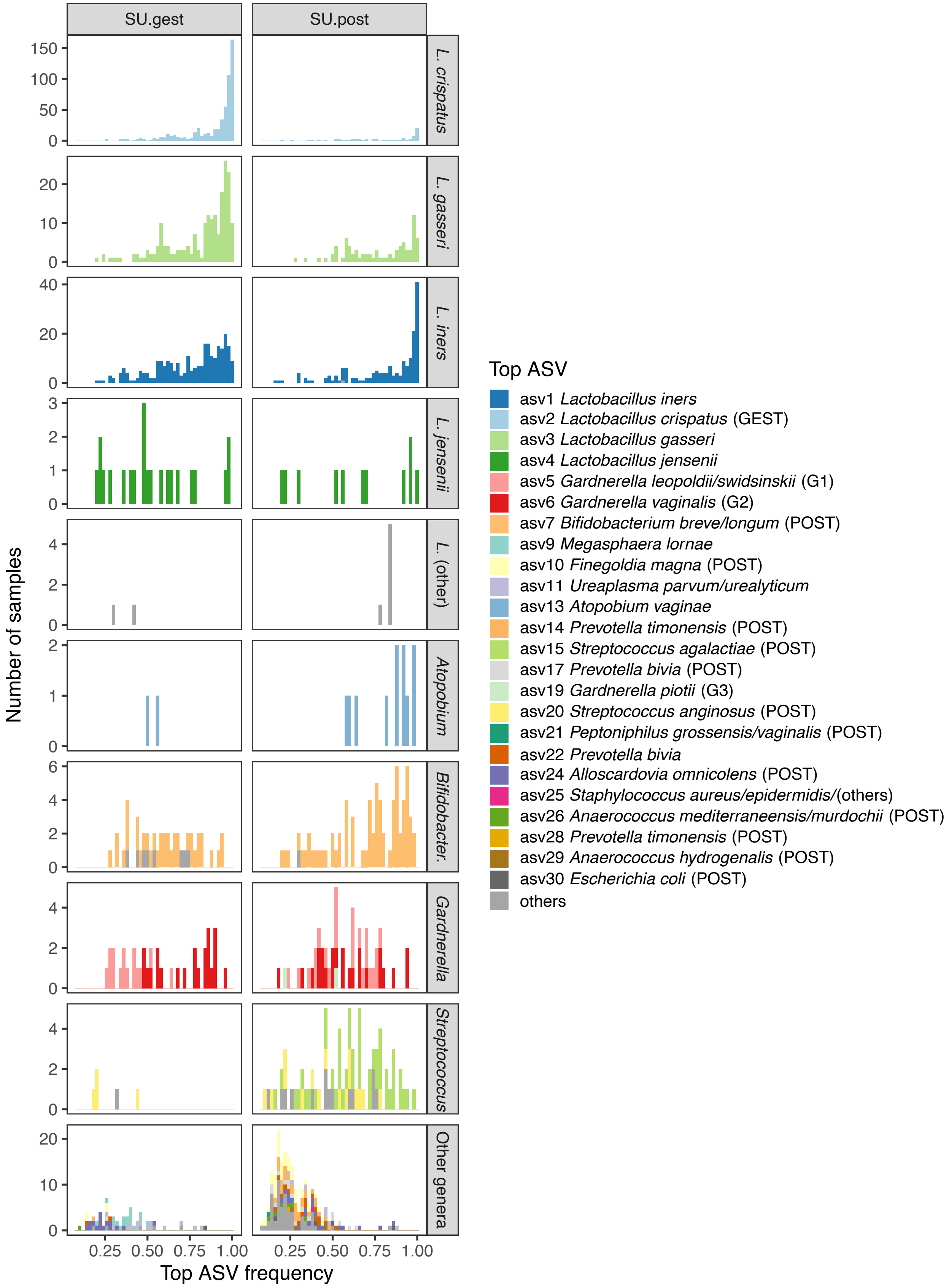

Supplementary Figure 7: Pre- and post-delivery vaginal bacterial communities viewed through the lens of each sample's most abundant ASV.

Histograms of top ASV frequency, which is defined as the relative abundance of the most abundant ASV in each sample. Samples are colored by the identity of the top ASV and faceted into rows by the taxonomy of the top ASV. The x-axis bin width is 0.02. Note that the y-axis scale differs among taxa. The figure depicts all samples collected from pregnancies in the postpartum subset (n = 72 SU pregnancies), with gestational samples displayed in left facets (SU.gest; n = 1195 unique samples) and postpartum samples displayed in right facets (SU.post; n = 745 unique samples). Twenty-four of the 30 most abundant ASVs study-wide, listed at right, appear as a top ASV in a sample collected from the postpartum subset. These 24 ASVs account for the top ASV in 95% of the depicted samples, in which they represent 86% of the total reads. For each of the 24 ASVs, we tested for an effect of phase (GEST versus POST) on the average frequency (the mean frequency across all unique samples from the same pregnancy). Those annotated in the legend were significantly more abundant in the given phase (two-sided Wilcoxon signed rank tests; phase indicated if effect size (r) > 0.2 and Benjamini-Hochberg-adjusted $P < 0.01$; n = 72 GEST phase versus n = 72 POST phase).

Supplementary Figure 8: Postpartum time-to-*Lactobacillus*-dominance depends on host and microbiota.

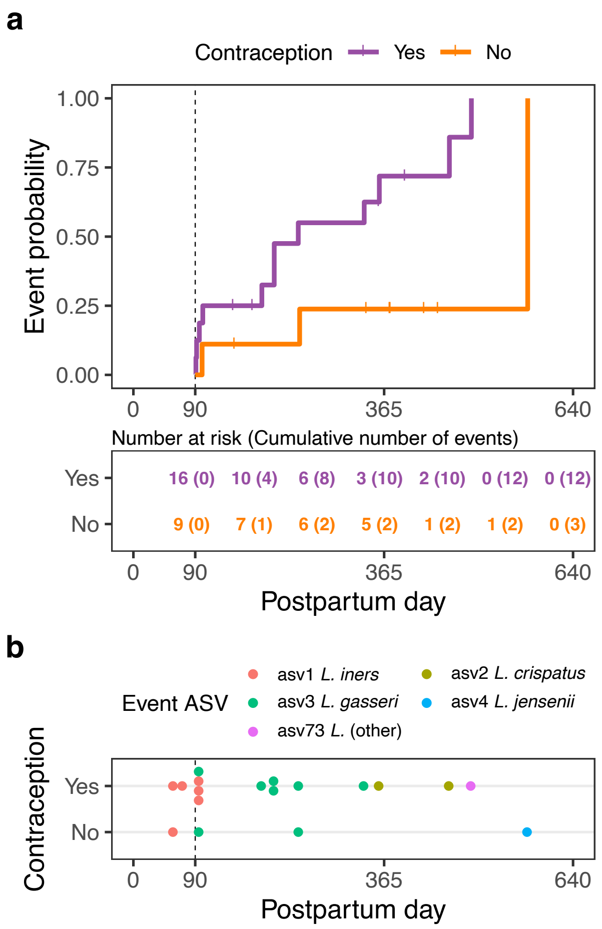

Supplementary Figure 8: Postpartum time-to-*Lactobacillus*-dominance depends on host and microbiota.

a, Landmarked analysis stratified by contraception use. Of 58 at-risk cases, 28 provided data for postpartum contraception (Supplementary Table 4). We found that the probability of *Lactobacillus* dominance was increased in those initiating contraception (any type; typically, prior to 90 days postpartum) compared to those who never initiated. Cox regression hazard ratio (HR) 4.82; 95% CI [1.07, 21.71]; $P = 0.04$; $n = 25$ cases. Three cases were excluded owing to a pre-landmark event. Tick marks indicate censored cases.

b, Dot plot identifying the dominant ASV for each of the 15 events depicted in panel **a**. The three pre-landmark events are also shown. The x-axis bin width is 14 days. The plot suggests that contraception use favors no single species, but that the identity of the dominant species may depend on the timing of initial dominance (explored further in Fig. 2c and Fig. 4c).

Supplementary Figure 9: Strong correlation between technical replicates exemplified using the Shannon diversity index, the frequency of *Lactobacillus crispatus*, and the frequency of *Lactobacillus iners*.

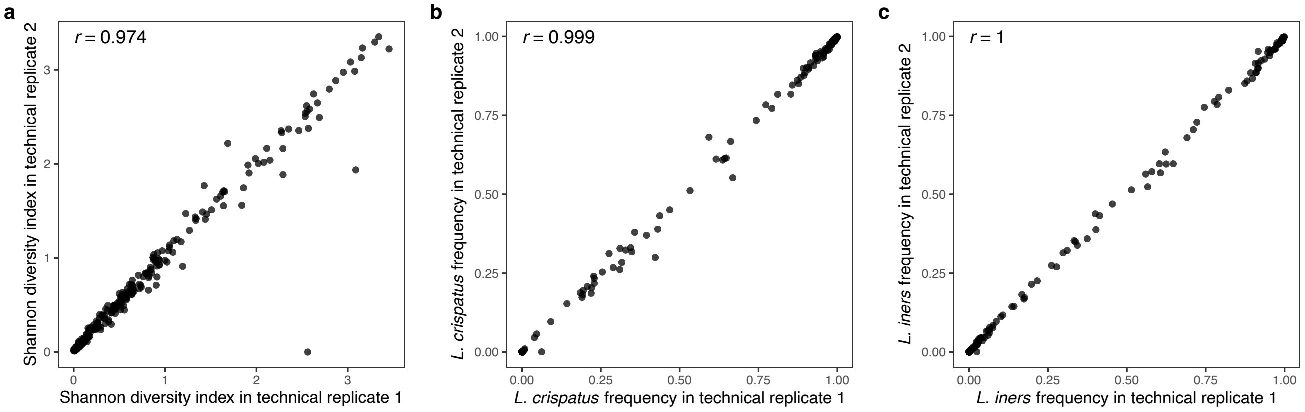

Supplementary Figure 9: Strong correlation between technical replicates exemplified using the Shannon diversity index, the frequency of *Lactobacillus crispatus*, and the frequency of *Lactobacillus iners*.

For a set of genomic DNA samples representing $n = 273$ unique vaginal swabs, amplification and sequencing took place on two separate occasions. These pairs of technical replicates were highly correlated for **a,** the Shannon diversity index; **b,** the frequency of *L. crispatus*; and **c,** the frequency of *L. iners*. Upper left, Pearson's product moment correlation coefficient.

Supplementary Tables

Supplementary Table 1. Demographic characteristics of study participants (*n* = 178) enrolled at Stanford University (SU) and the University of Alabama at Birmingham (UAB)

	SU		UAB
Characteristic	Full cohort (*n* = 82)	Postpartum subset (*n* = 59)	Full cohort (*n* = 96)
Median age at enrollment, years (range)	32 (25 - 43)	31 (25 - 43)	25 (17 – 38)
Race:			
American Indian	1 (1%)	1 (2%)	0
Asian	20 (24%)	18 (31%)	1 (1%)
Black	4 (5%)	4 (7%)	80 (83%)
Other	5 (6%)	4 (7%)	4 (4%)
Pacific Islander	0	0	1 (1%)
White	51 (62%)	32 (54%)	9 (9%)
Declined to state	1 (1%)	0	1 (1%)
Ethnicity:			
Hispanic	13 (16%)	5 (8%)	5 (5%)
Not Hispanic	69 (84%)	54 (92%)	89 (93%)
Declined to state	0	0	2 (2%)
Median pre-pregnancy BMI, kg/m^2 (range)	22.5 (17.3 – 50.5)	22.0 (17.3 – 50.5)	28.0 (15.8 - 73.0)

BMI, body mass index

Supplementary Table 2. Clinical characteristics of participants' pregnancies (*n* = 196) enrolled at Stanford University (SU) and the University of Alabama at Birmingham (UAB)

	SU		UAB
Characteristic	Full cohort (*n* = 100)	Postpartum subset (*n* = 72)	Full cohort (*n* = 96)
Nulliparous	42 (42%)	32 (44%)	2 (2%)
History of prior live birth	55 (55%)	38 (53%)	93 (97%)
History of prior preterm birth	19 (19%)	11 (15%)	88 (92%)
Median birth-to-conception interval, months (range)	25 (4 – 183)	20 (5 – 183)	31 (2 – 158)
Outcome:			
Miscarriage	2 (2%)	0	0
Preterm delivery	11 (11%)	5 (7%)	41 (43%)
Term delivery	87 (87%)	67 (93%)	55 (57%)
Median gestational age at delivery, days (range)	275 (219 – 293)	275 (230 – 292)	260 (123 – 289)
Median maternal age at delivery, years (range)	33.0 (26.0 – 43.7)	33.0 (26.0 – 43.7)	26.5 (17.6 – 39.1)
Spontaneous labor onset	39 (40%)	29 (40%)	66 (69%)
Spontaneous rupture of membranes (SROM)	31 (32%)	21 (29%)	59 (61%)
GBS status, neg/pos/unk	75/21/2	56/14/2	48/17/31
Intrapartum antibiotics	45 (46%)	33 (46%)	48 (50%)
Cesarean delivery	30 (31%)	19 (26%)	22 (23%)
Female baby	46 (47%)	33 (46%)	40 (42%)

GBS, group B *Streptococcus*

Supplementary Table 3. Association between delivery and ASVs in pairs of samples immediately flanking delivery (SU cohort, *n* = 70 pregnancies)

ASV	Change in (mean rank in) relative abundance	Effect size (*r*)	Significance code
asv2 *Lactobacillus crispatus*	decreased	0.63	****
asv4 *Lactobacillus jensenii*	decreased	0.51	****
asv3 *Lactobacillus gasseri*	decreased	0.42	***
asv1 *Lactobacillus iners*	decreased	0.28	*
asv11 *Ureaplasma parvum*/*urealyticum*	decreased	0.27	*
asv82 *Actinomyces neuii*	decreased	0.08	ns
asv25 *Staphylococcus aureus*/*epidermidis*/(others)	decreased	0.05	ns
asv43 *Prevotella buccalis*	increased	0.75	****
asv21 *Peptoniphilus grossensis*/*vaginalis*	increased	0.72	****
asv38 *Dialister propionicifaciens*	increased	0.70	****
asv77 *Ezakiella coagulans*	increased	0.69	****
asv26 *Anaerococcus mediterraneensis*/*murdochii*	increased	0.67	****
asv115 *Peptoniphilus lacrimalis*	increased	0.67	****
asv84 *Anaerococcus vaginalis*	increased	0.66	****
asv133 *Peptoniphilus urinimassiliensis*	increased	0.66	****
asv33 *Campylobacter ureolyticus*	increased	0.65	****
asv100 *Clostridiales* KU726662	increased	0.65	****
asv14 *Prevotella timonensis*	increased	0.64	****
asv64 *Peptoniphilus duerdenii*	increased	0.64	****
asv102 *Porphyromonas asaccharolytica*	increased	0.64	****
asv10 *Finegoldia magna*	increased	0.64	****
asv88 *Porphyromonas uenonis*	increased	0.62	****
asv59 *Peptoniphilus gorbachii*/*harei*/*lacydonensis*/*tyrrelliae*	increased	0.61	****
asv106 *Mobiluncus curtisii*	increased	0.60	****
asv81 *Peptoniphilus coxii*	increased	0.59	****
asv36 *Lawsonella clevelandensis*	increased	0.58	****
asv29 *Anaerococcus hydrogenalis*	increased	0.57	****
asv60 *Varibaculum anthropi*/*cambriense*	increased	0.57	****
asv87 *Campylobacter hominis*	increased	0.56	****
asv47 *Fenollaria massiliensis*	increased	0.56	****
asv93 *Porphyromonas bennonis*	increased	0.53	****
asv39 *Anaerococcus obesiensis*	increased	0.53	****
asv32 *Corynebacterium amycolatum*/*lactis*/*xerosis*	increased	0.53	****
asv52 *Dialister micraerophilus*	increased	0.52	****
asv56 *Anaerococcus prevotii*/*tetradius*	increased	0.50	***
asv17 *Prevotella bivia*	increased	0.48	***
asv54 *Fusobacterium nucleatum*	increased	0.45	***
asv28 *Prevotella timonensis*	increased	0.42	***
asv42 *Prevotella disiens*	increased	0.38	**
asv31 *Corynebacterium tuberculostearicum*	increased	0.34	**
asv20 *Streptococcus anginosus*	increased	0.32	**
asv91 *Anaerococcus senegalensis*	increased	0.27	*
asv62 *Corynebacterium pseudogenitalium*	increased	0.26	*
asv78 *Corynebacterium pyruviciproducens*	increased	0.23	*
asv22 *Prevotella bivia*	increased	0.23	*

Test performed if ASV had a relative abundance > 0.001 in at least 15% of tested samples (*n* = 45 ASVs; all shown). Significance levels correspond to Benjamini-Hochberg-adjusted *P* values for paired, two-sided Wilcoxon signed rank tests. ****, P < 0.0001; ***, P < 0.001; **, P < 0.01; *, P < 0.05; ns, not significant

Supplementary Table 4. Postpartum contraception methods

Method	SU Postpartum subset (n = 72)
Birth control implant	2
Birth control injection	1
Birth control patch	1
Birth control pill	3
IUD, copper	2
IUD, progestin	2
IUD, unspecified type	6
Condoms	2
Tubal ligation	1
None	12
Did not respond	40

IUD, Intrauterine device
