# Supplementary material for: Abrupt perturbation and delayed recovery of the vaginal ecosystem following childbirth
Source: Nat Commun. 2023 Jul 12;14:4141. doi: 10.1038/s41467-023-39849-9 (PMC10338445; doi:10.1038/s41467-023-39849-9)
Supplement: Supplementary file 3 — Reporting Summary [file 41467_2023_39849_MOESM3_ESM.pdf]

nature portfolio

Corresponding author(s): Elizabeth Costello & David Relman

Last updated by author(s): Jun 22, 2023

Reporting Summary

Nature Portfolio wishes to improve the reproducibility of the work that we publish. This form provides structure for consistency and transparency in reporting. For further information on Nature Portfolio policies, see our Editorial Policies and the Editorial Policy Checklist.

Statistics

For all statistical analyses, confirm that the following items are present in the figure legend, table legend, main text, or Methods section.

n/a	Confirmed	
☐	☒	The exact sample size (n) for each experimental group/condition, given as a discrete number and unit of measurement
☐	☒	A statement on whether measurements were taken from distinct samples or whether the same sample was measured repeatedly
☐	☒	The statistical test(s) used AND whether they are one- or two-sided<br>*Only common tests should be described solely by name; describe more complex techniques in the Methods section.*
☐	☒	A description of all covariates tested
☐	☒	A description of any assumptions or corrections, such as tests of normality and adjustment for multiple comparisons
☐	☒	A full description of the statistical parameters including central tendency (e.g. means) or other basic estimates (e.g. regression coefficient) AND variation (e.g. standard deviation) or associated estimates of uncertainty (e.g. confidence intervals)
☐	☒	For null hypothesis testing, the test statistic (e.g. F, t, r) with confidence intervals, effect sizes, degrees of freedom and P value noted<br>*Give P values as exact values whenever suitable.*
☒	☐	For Bayesian analysis, information on the choice of priors and Markov chain Monte Carlo settings
☒	☐	For hierarchical and complex designs, identification of the appropriate level for tests and full reporting of outcomes
☐	☒	Estimates of effect sizes (e.g. Cohen's d, Pearson's r), indicating how they were calculated

Our web collection on statistics for biologists contains articles on many of the points above.

Software and code

Policy information about availability of computer code

Data collection	No special software was used during data collection.
Data analysis	Analysis code and data are available at the Stanford Digital Repository: https://purl.stanford.edu/pz745bc9128<br>We used open source software QIIME1 (v.1.9.1), QIIME2 (v.2018.2), cutadapt (v.1.14), dada2 (v.1.8), phyloseq (v.1.34.0), vsearch (v.2.15.1), R (v.4.0.4), RStudio (v.1.4.1717), vegan (v.2.5.7), nlme (v.3.1.153), sandwich (v.3.0.1), survival (v.3.2.13), survminer (v.0.4.9), ALDEx2 (v.1.22.0), treeDA (v.0.0.5), rmcorr (v.0.4.4), igraph (v.1.2.8), ggtree (v.3.3.0.900), ggpubr (v.0.4.0), rstatix (v.0.7.0), tidyverse (v.1.3.1) and magrittr (v.2.0.1). We also used a taxonomy and backbone tree based on the Silva reference database (v.132).

For manuscripts utilizing custom algorithms or software that are central to the research but not yet described in published literature, software must be made available to editors and reviewers. We strongly encourage code deposition in a community repository (e.g. GitHub). See the Nature Portfolio guidelines for submitting code & software for further information.

Data

Policy information about availability of data

All manuscripts must include a data availability statement. This statement should provide the following information, where applicable:

- Accession codes, unique identifiers, or web links for publicly available datasets
- A description of any restrictions on data availability
- For clinical datasets or third party data, please ensure that the statement adheres to our policy

Raw sequencing reads were deposited in NCBI's Sequence Read Archive under BioProjects PRJNA393472 and PRJNA821262.

Human research participants

Policy information about studies involving human research participants and Sex and Gender in Research.

Reporting on sex and gender	Our study was of pregnancy, childbirth, and the postpartum period. We enrolled people who were pregnant, had been pregnant, or were trying to become pregnant.
Population characteristics	We enrolled healthy individuals of child-bearing age from the population served by Lucile Packard Children's Hospital at Stanford University (San Francisco Bay Area, California, USA). These participants ranged in age from 25-43 years old. We also re-analyzed previously published data from a cohort of pregnant individuals enrolled at the University of Alabama at Birmingham (Alabama, USA). These participants ranged in age from 17-38 years old, were at high risk for preterm birth, and were treated with intramuscular 17α-hydroxyprogesterone caproate. Further details can be found in Supplementary Table 1.
Recruitment	Individuals presenting for prenatal care or preconception consultation at the obstetrics clinics of Lucile Packard Children's Hospital at Stanford were invited to participate. Inclusion criteria were age 18 years or older, singleton gestation, not immunosuppressed, able to perform the study procedures, and able to provide written informed consent. Participants electing to continue participation after the end of a pregnancy or to resume participation at the start of a subsequent pregnancy were re-enrolled. The subset of pregnancies followed postpartum was slightly enriched for term deliveries (93%) relative to the Stanford University cohort as a whole (87%; Supplementary Table 2).
Ethics oversight	The study was approved by the Institutional Review Boards of Stanford University (protocol no. 21956) and the University of Alabama at Birmingham (protocol no. X121031002). All subjects provided written informed consent before completing an enrollment questionnaire and providing biological samples.

Note that full information on the approval of the study protocol must also be provided in the manuscript.

Field-specific reporting

Please select the one below that is the best fit for your research. If you are not sure, read the appropriate sections before making your selection.

☐ Life sciences ☐ Behavioural & social sciences ☒ Ecological, evolutionary & environmental sciences

For a reference copy of the document with all sections, see nature.com/documents/nr-reporting-summary-flat.pdf

Ecological, evolutionary & environmental sciences study design

All studies must disclose on these points even when the disclosure is negative.

Study description	A longitudinal study of the human vaginal ecosystem before and after childbirth. The study comprises 3,848 unique vaginal swabs and represents longitudinal sampling of 82 SU-cohort participants over 100 pregnancies and 96 UAB-cohort participants over 96 pregnancies. Among cases with at least one post-delivery sample available for analysis (72 SU-cohort pregnancies), 93% delivered at term and the median extent of monthly postpartum sampling was 365 days [interquartile range (IQR) 332-375 days; range 6-790 days]. The study was observational in nature. There was no intervention.
Research sample	As described above, we enrolled healthy individuals of child-bearing age from the population served by Lucile Packard Children's Hospital at Stanford (San Francisco Bay Area, California, USA). We also re-analyzed previously published data from a cohort of pregnant individuals enrolled at the University of Alabama at Birmingham. These data were sourced from the Stanford Digital Repository: https://purl.stanford.edu/yb681vm1809. Our study was of pregnancy, childbirth, and the postpartum period. We enrolled people who were pregnant, had been pregnant, or were trying to become pregnant. The participants ranged in age from 17 to 43 years old at enrollment. The cohorts were meant to represent the populations of North American women residing in the geographic areas served by the two hospitals involved in enrollment.
Sampling strategy	As described above, individuals presenting for prenatal care or preconception consultation at the obstetrics clinics of Lucile Packard Children's Hospital at Stanford were invited to participate. Inclusion criteria were age 18 years or older, singleton gestation, not immunosuppressed, able to perform the study procedures, and able to provide written informed consent. Subjects electing to continue participation after the end of a pregnancy or to resume participation at the start of a subsequent pregnancy were re-

	enrolled. The sample size was deemed sufficient to capture relevant dynamics occurring over the year following delivery. No sample size calculation was performed.
Data collection	Demographic and clinical data, including reproductive histories, were collected in a detailed questionnaire at enrollment and in brief follow-up questionnaires at prenatal visits (staff in the obstetrical clinics and the labor and delivery unit of Lucile Packard Children's Hospital; study coordinators at the March of Dimes Prematurity Research Center at Stanford; Stevenson lab). Mid-vaginal swabs were self-collected by the participants. Following transport to the laboratory, the swabs were stored at -80°C until further processing (study coordinators at the March of Dimes Prematurity Research Center at Stanford; Stevenson lab). Swabs were analyzed for microbiota composition using 16S rRNA gene amplicon sequencing (Relman lab) and for human cytokines/chemokines using a multiplexed bead assay (Kwon lab). Sequencing was performed at the DNA Services Lab, Roy J. Carver Biotechnology Center, University of Illinois at Urbana-Champaign.
Timing and spatial scale	Sample collection occurred on a weekly basis from enrollment to delivery, and on a monthly basis from delivery to a maximum of 36 months postpartum or the start of a next pregnancy. Participants were asked to collect a first postpartum sample at or around six weeks after delivery. The samples analyzed in this study were collected between November 2011 and September 2018. The data are taken from the spatial scale of a mid-vaginal swab. The spatial scale of a mid-vaginal swab is likely on the order of several inches.
Data exclusions	A small number of samples failed PCR amplification for unknown reasons, resulting in low sequencing yield (< 40k reads per sample). These samples were excluded from most analyses.
Reproducibility	The study was observational. There were no experiments to reproduce.
Randomization	Randomization was not relevant to this study because participants were not allocated into groups.
Blinding	Blinding was not relevant to this study because it did not include a treatment group.

Did the study involve field work? ☐ Yes ☒ No

Reporting for specific materials, systems and methods

We require information from authors about some types of materials, experimental systems and methods used in many studies. Here, indicate whether each material, system or method listed is relevant to your study. If you are not sure if a list item applies to your research, read the appropriate section before selecting a response.

Materials & experimental systems

n/a	Involved in the study
☒	☐ Antibodies
☒	☐ Eukaryotic cell lines
☒	☐ Palaeontology and archaeology
☒	☐ Animals and other organisms
☒	☐ Clinical data
☒	☐ Dual use research of concern

Methods

n/a	Involved in the study
☒	☐ ChIP-seq
☒	☐ Flow cytometry
☒	☐ MRI-based neuroimaging
